# Advances in *Cryptococcus* genomics: insights into the evolution of pathogenesis

**DOI:** 10.1590/0074-02760170473

**Published:** 2018-02-19

**Authors:** Christina A Cuomo, Johanna Rhodes, Christopher A Desjardins

**Affiliations:** 1 Broad Institute of Massachusetts; Institute of Technology and Harvard, Cambridge, MA, USA; 2 Department of Infectious Disease Epidemiology, Imperial College London, London, United Kingdom

**Keywords:** Cryptococcus neoformans, Cryptococcus gattii, MLST, genome, sequencing, evolution, recombination

## Abstract

*Cryptococcus* species are the causative agents of cryptococcal meningitis, a significant source of mortality in immunocompromised individuals. Initial work on the molecular epidemiology of this fungal pathogen utilized genotyping approaches to describe the genetic diversity and biogeography of two species, *Cryptococcus neoformans* and *Cryptococcus gattii*. Whole genome sequencing of representatives of both species resulted in reference assemblies enabling a wide array of downstream studies and genomic resources. With the increasing availability of whole genome sequencing, both species have now had hundreds of individual isolates sequenced, providing fine-scale insight into the evolution and diversification of *Cryptococcus* and allowing for the first genome-wide association studies to identify genetic variants associated with human virulence. Sequencing has also begun to examine the microevolution of isolates during prolonged infection and to identify variants specific to outbreak lineages, highlighting the potential role of hyper-mutation in evolving within short time scales. We can anticipate that further advances in sequencing technology and sequencing microbial genomes at scale, including metagenomics approaches, will continue to refine our view of how the evolution of *Cryptococcus* drives its success as a pathogen.

The genome sequencing of *Cryptococcus*, as for many fungi, initially focused on generating assemblies of a few reference isolates (Cuomo & Birren 2010, Cuomo 2017). These assemblies provided high quality representations of the genome, including some that consist of chromosomal size scaffolds; however, due to difficulties in sequencing and assembling some genomic regions, gaps remained typically at centromeres or chromosome ends and occasionally at other internal regions. With the early release of this genomic data, the availability of the nearly complete catalog of genes for these species were rapidly utilized in studies of individual genes, gene families and biological pathways; the sequence was also leveraged to build functional genomic resources.

Recent years have seen dramatic expansion in the scale of fungal genome sequencing, with the falling cost and increased availability of sequencing technologies. This includes annotated genome assemblies representing each of the varieties of *C. neoformans*, var. *grubii* and var. *neoformans*, and each of the four major monophyletic lineages of *C. gattii* (VGI, VGII, VGIII, and VGIV) (Table) (Loftus et al. 2005, D’Souza et al. 2011, Janbon et al. 2014, Farrer et al. 2015, Rhodes et al. 2017b). Each of these six subdivisions and one additional novel group were proposed to be separate species and assigned new names (Table), though this revision of the taxonomy is debated (Hagen et al. 2015, Kwon-Chung et al. 2017). While *Cryptococcus* isolates are primarily haploid, diploid hybrid isolates of *C. neoformans* var. *grubii* and *C. neoformans* var. *neoformans* have also been reported (Table) (Franzot et al. 1999, Desnos-Ollivier et al. 2015). An increasing number of large scale studies take a re-sequencing approach utilizing high quality reference genome assemblies to detect variants across hundreds of genomes, in many cases leveraging prior multilocus sequence typing (MLST) data to select isolates. Comparing genomes at this scale has provided a fine scale view of the genetic diversity and level of exchange between lineages and across geographic regions, characterized how gene gain and loss have impacted these species and lineages, and enabled genome-wide association studies of phenotypes related to virulence. Here, we review the progress in genome sequencing of *Cryptococcus* and highlight how increasing scale has been applied to examine both macro and microevolution of both *C. neoformans* and *C. gattii*.

**TABLE:** Major subdivisions within the *Cryptococcus neoformans* and *Cryptococcus gattii* species

Species	Variety	Molecular Type	AFLP	Proposed species name
*C. neoformans*	var. *grubii*	VNI VNII VNB	AFLP1	*C. neoformans*
	var. *neoformans*	VNIV	AFLP2	*C. deneoformans*
	AD hybrid	VNII	AFLP3	*C. neoformans* x *C. deneoformans* hybrid
*C. gattii*		VGI	AFLP4	*C. gattii*
		VGII (a, b and c sub-genotypes)	AFLP6	*C. deuterogattii*
		VGIII (a, b and c sub-genotypes)	AFLP5	*C. bacillisporus*
		VGIV	AFLP7	*C. tetragattii*
		VGIV/VGIIIc	AFLP10	*C. decagattii*

*Population studies by MLST* - While a number of genotyping methods have been used to study *C. neoformans* var. *grubii* and *C. gattii*, in 2009 the International Society for Human and Animal Mycology (ISHAM) *Cryptococcus* Working Group agreed upon MLST as the standardized genotyping approach. MLST can have high discriminatory power with appropriately selected loci, give reproducible results between laboratories, and has been applied to differentiate species and to study the epidemiology of fungal pathogens (Taylor & Fisher 2003). Seven loci, comprised of six protein coding genes (*CAP59*, *GPD1*, *LAC1*, *PLB1*, *SOD1* and *URA5*) and the intergenic spacer IGS1 (Meyer et al. 2009), were selected to identify all eight major molecular types of both species. The resulting allele types are assigned according to either Litvintseva et al. (2006) for *C. neoformans* var. *grubii*, or Fraser et al. (2005) for *C. gattii*.

MLST analysis revealed subpopulations within *C. neoformans* var. *grubii* and *C. gattii* (Litvintseva et al. 2006, Bovers et al. 2008, Ngamskulrungroj et al. 2009) and subsequently identified fine-grain geographical associations with particular sequence types (Chowdhary et al. 2011, Beale et al. 2015, Lam et al. 2017). In providing initial estimates of variation within the population, higher estimates of diversity in South African *C. neoformans* var. *grubii* isolates led to the hypothesis that this population was ancestral and isolates spread around the globe ‘out of Africa’ (Litvintseva et al. 2011, Simwami et al. 2011, Litvintseva & Mitchell 2012). By contrast, high similarity of MLST profiles can suggest more recent transitions, such as the apparent migration of the VNI lineage from African populations to Asia, infecting predominantly to HIV-positive patients (Simwami et al. 2011, Khayhan et al. 2013). Similar MLST analyses identified high diversity of the *C. gattii* VGII lineage in northern Brazil, suggesting this location could be the source for the global expansion of VGII including the outbreak in the Pacific Northwest (Hagen et al. 2013, Souto et al. 2016). These comparisons also revealed the introduction of Australian VGII into Vancouver Island, Canada (Fraser et al. 2005, Byrnes 3rd et al. 2009, 2010). Thus, these MLST studies were fundamental in describing the population structure of these species and describing associations with geography.

A limitation of MLST is that only a small fraction of the sequence diversity is captured (Beale et al. 2015) and this may provide incomplete or in some cases inaccurate measures of the species relationships. More specifically, comparing inadequate data and relying on congruence can lead to incorrect assertions of recombination (Tibayrenc & Ayala 2014) and of the relationships of isolates, as most phylogenetic methods rely on an assumption of vertical descent. Some of the *Cryptococcus* MLST loci have been shown to have undergone recombination (Litvintseva et al. 2006) and therefore including them among a small number of loci in a multi-gene phylogeny may result in an inaccurate view of the species-level relationships of some isolates.

*Reference genome sequencing projects* - The earliest completed fungal genomes, sequenced using Sanger technology, included several *Cryptococcus* isolates (Galagan et al. 2005, Cuomo & Birren 2010). The first report described the genomes of two related isolates of *C. neoformans* var. *neoformans*, JEC21 and B-3501 (Loftus et al. 2005). Several years later, the genomes of two isolates of *C. gattii* were compared; WM276 was selected to represent the predominant VGI lineage and R265 to represent the VGII lineage responsible for an outbreak in the Pacific Northwest (Fraser et al. 2005, D’Souza et al. 2011). The genome for the H99 VNI isolate of *C. neoformans* var. *grubii* was the first to incorporate RNA-Seq, generated from yeast grown in a variety of conditions, to predict a deep catalog of alternatively spliced transcripts and non-coding genes (Janbon et al. 2014). More recently, high quality reference assemblies for larger numbers of isolates have been generated from Illumina sequencing. To date, 6 *C. gattii*, 3 *C. neoformans* var. *neoformans*, and 49 *C. neoformans* var. *grubii* assemblies have been deposited onto NCBI. These annotated genomes include representatives of all the major molecular types of *C. gattii* (including VGIII and VGIV isolates) and *C. neoformans* var. *grubii* (including VNII and VNB isolates) (Farrer et al. 2015, Rhodes et al. 2017a).

Overall genome structure was found to be highly conserved across *Cryptococcus* with a small number of rearrangements detected between species or molecular types. The chromosomal sequence of isolates of the different molecular types of *C. gattii* were found to be highly co-linear, with a small number of rearrangements detected; sequence divergence of ~7% on average (D’Souza et al. 2011, Farrer et al. 2015) supported a suggestion that these could represent different species (D’Souza et al. 2011). The genomes of *C. neoformans* var. *grubii* and *C. neoformans* var. *neoformans* also vary in structure, with three translocations and two large inversions; more extensive rearrangements are observed for these genomes in comparison to *C. gattii* (Janbon et al. 2014).

Characterization of gene content and structure revealed differences in *Cryptococcus* compared to other fungi. *Cryptococcus* genes are intron-rich (Loftus et al. 2005) with extensive alternative splicing predicted from RNA-Seq (Janbon et al. 2014). While gene content is highly conserved in *C. gattii*, variable genes are enriched for specific functions including response to oxidative stress, mitochondrial import, and metal binding and transport (Farrer et al. 2015). One notable difference between the *C. gattii* molecular types involves the loss of genes involved in RNA interference in VGII isolates (D’Souza et al. 2011, Farrer et al. 2015). Variation in gene content has also been examined *in C. neoformans* var. *grubii*; a recent study assembled and annotated 39 genomes representing each of the major lineages (VNI, VNII, and VNB) (Rhodes et al. 2017b). A small number of genes specific to each lineage were identified, including transporters and transcription factors; these functional categories were also found to include the most rapidly evolving genes (Rhodes et al. 2017b).

The sequenced reference genomes have also enabled the construction of large-scale functional genomic resources. A deletion collection of all *C. neoformans* var. *grubii* genes in the H99 strain background is underway (Liu et al. 2008), which helps support routine analysis of the requirement for specific genes under different conditions. Smaller functionally-focused deletion collections of transcription factors (Jung et al. 2015) and kinases (Lee et al. 2016) have already been completed and analyzed, making a host of new connections between specific genes and virulence or virulence-related phenotypes.

*Population sequencing of C. gattii* - Building on the analysis of reference genomes and assemblies, several studies have examined genetic diversity in *C. gattii* using whole genome sequencing. Available genome sequence represents four major molecular types of *C. gattii* (VGI, VGII, VGIII, and VGIV) and some more rarely observed clades related to these lineages. Isolates of VGII have been well sampled by sequencing to understand the recent outbreak in the Pacific Northwest. In addition, VGIII has been deeply sequenced in an effort to characterize an increasing number of infections in the USA, South and Central America. By contrast, the VGI and VGIV groups are not as deeply sequenced to date; VGI isolates are common to Asia, Australia, and Europe, whereas VGIV is most prevalent in India and Africa.

Initial studies of VGII diversity examined the relationship of isolates in the outbreak lineages in the Pacific Northwest to those found in other geographic regions. In 1999, an outbreak of *C. gattii* occurred on Vancouver Island in Canada and expanded to the Pacific Northwest, a region outside the typically tropical and subtropical range of this pathogen (Hoang et al. 2004, Fraser et al. 2005). These studies confirmed that there are three major subgroups of VGII, including the VGIIa and VGIIc lineages that are largely restricted to the Pacific northwest and the more widely distributed VGIIb (Billmyre et al. 2014, Engelthaler et al. 2014). Using phylogenetic methods, South American isolates were placed as outgroups to both VGIIa and VGIIb (Billmyre et al. 2014, Engelthaler et al. 2014); in addition, novel VGII genotypes outside these major subgroups most frequently include isolates from South America (Engelthaler et al. 2014), supporting an origin of South America for this lineage as also suggested by aforementioned MLST studies (Hagen et al. 2013, Souto et al. 2016). While the major subgroups each appear highly clonal, as they have one mating type and no evidence of recombination, there is evidence of ancestral recombination between the subgroups (Billmyre et al. 2014, Engelthaler et al. 2014). Examining mutations specific to the VGIIa outbreak lineage revealed a frameshift mutation in an ortholog of the *MSH2* DNA mismatch repair gene; this was found only in the VGIIa-like isolates which are lower in virulence than other VGIIa isolates (Billmyre et al. 2014). The rate of mutation was higher in homopolymer runs in the VGIIa-like isolates; however, these were not detected in other VGIIa outbreak lineages or ancestral branches, suggesting that loss of *MSH2* and higher mutation rate and may not have played a role in adaption in the virulent isolates within the outbreak lineage (Billmyre et al. 2017).

Recent studies have characterized the subdivisions and geographical associations of isolates in the VGIII group by comparing genome sequences. In Southern California, *C. gattii* infections of HIV/AIDS patients are predominantly caused by VGIII isolates (Byrnes 3rd et al. 2011); isolation of local environmental isolates revealed a very close relationship, with three pairs of clinical and environmental isolates each separated by an average of only 106 SNPs, suggesting a local environmental reservoir of *C. gattii* VGIII (Springer et al. 2014). MLST study of VGIII isolates revealed two separated subgroups, VGIIIa and VGIIIb (Byrnes 3rd et al. 2011), corresponding to two different serotypes (Firacative et al. 2016). A wider study of 60 VGIII genomes supported these two clades and notably identified two small related clades of VGIII-like isolates, one of which shares most MLST alleles with the AFLP10/*C. decagattii* isolate (Hagen et al. 2015, Firacative et al. 2016). Further analyses of the sequenced genomes of VGIIIa, VGIIIb, and AFLP10/*C. decagattii* isolates are needed to characterize the level of genetic exchange between these groups.

Farrer et al. (2015) combined population-level sequencing of 37 isolates with 16 reference assemblies of *C. gattii*, and identified a new transcontinental sub-lineage of VGII, named VGIIx. Comparison of the mitochondrial and nuclear genomes revealed evidence of recombination between the VGII and VGIII mitochondrial but not nuclear genomes, providing a glimpse of genetic exchange processes within *C. gattii.* Furthermore, tests of selection between all major lineages and sub-lineages of *C. gattii* identified multidrug transporters, aconitases (iron-sulfur proteins), capsule genes, heat-shock proteins and protein kinases under positive selection in multiple sub-lineages (Farrer et al. 2015, 2016).

*Population sequencing of C. neoformans* - A number of recently published population-level studies have greatly expanded the knowledge of genomic diversity of *C. neoformans* var. *grubii* available (Desjardins et al. 2017, Rhodes et al. 2017b, Vanhove et al. 2017). These studies recapitulated the three traditional lineages (VNI, VNII, and VNB) but also supported splitting VNB into two lineages, VNBI and VNBII, a pattern that had been noted previously in AFLP- and MLST-based studies (Litvintseva et al. 2003, 2006, Chen et al. 2015). Recombination was found to be occurring within all tested lineages including the largely unisexual VNI, although at a slightly slower rate than VNBI and VNBII (Desjardins et al. 2017). Recombination between the three lineages appeared limited, suggesting some degree of reproductive isolation. A number of inter-lineage hybrids were identified, however, including some that likely resulted from recent meiotic crossing-over, as evidenced by large blocks of single-lineage ancestry across each chromosome (Rhodes et al. 2017b). Aneuploidy was found across all chromosomes in both single-lineage and hybrid strains, and a number of diploid hybrids between *C. neoformans* var. *grubii* and *C. neoformans* var. *neoformans* showed aneuploidy leading to loss-of-heterozygosity across various chromosomes. In sum, these analyses point to limited but definitive genetic exchange between the *C. neoformans* var. *grubii* lineages.

The division of VNB into VNBI and VNBII enabled characterization of notable phenotypic differences between these two groups. In Botswana, VNBII was enriched for clinical isolates relative to VNBI (Desjardins et al. 2017) and the same trend was seen in Zambian isolates (Vanhove et al. 2017). Surprisingly, high-throughput phenotyping showed that VNBI environmental isolates were more resistant to oxidative stress and more heavily melanized that VNBI clinical isolates, perhaps reflecting a greater breadth of selective pressures in the environment than in the human host (Desjardins et al. 2017).

The characterization of population structure and recombination levels also guided selection of isolates for genome-wide association studies. Within VNB, mutations in virulence factors and stress response genes were found to be associated with clinical isolates versus environmental, and loss-of-function mutations in the transcription *BZP4* linked to melanization capacity (Desjardins et al. 2017). Thus, analyses of these large-scale genomic data have begun to identify a host of potential virulence factors and mutations to be directly tested in the laboratory setting.

Sequencing isolates from poorly represented geographic regions has provided invaluable insight into the biogeography of *Cryptococcus*. Vanhove et al. (2017) found that *C. neoformans* var. *grubii* and *C. gattii* occupied different ecological niches, with the former primarily recovered from Zambezi Mopane woodlands and the latter primarily recovered from Central Miombo woodlands. Sequencing of a number of *C. neoformans* var. *grubii* clinical isolates from Brazil revealed that both the VNBI and VNBII lineages are naturally occurring there (Rhodes et al. 2017b). Phylogenomic analysis revealed that deep branches separated South American and African isolates in both VNBI and VNBII, suggesting that migration of these lineages between continents occurred prior to their diversification (Rhodes et al. 2017b). This suggests that the transcontinental spread of *C. neoformans* var. *grubii* probably occurred much earlier than previously thought.

*Serial isolates from patients and microevolution* - In contrast to macro-evolutionary patterns described with genomic studies of global populations, genome sequencing has also been leveraged to study micro-evolution of *Cryptococcus* within patients by comparing isolates at initial presentation and weeks later during relapse of infection (Ormerod et al. 2013, Chen et al. 2017, Rhodes et al. 2017a). Ormerod et al. (2013) sequenced a pair of initial and relapse isolates with marked virulence-related phenotypic differences (including growth rate at high temperature, capsule size, and melanization); a small number of genetic changes were identified by comparing variants, including deletion of a transcriptional regulator and changes in copy number on the arms of chromosome 12. Chen et al. (2017) sequenced 38 initial and relapse isolates from 18 patients and found mutations occurring in genes involved in a number of virulence-related phenotypes, including growth at 39ºC, stress response, and capsule production. They also identified a 13-fold amplification of a small region of chromosome 1 including *ERG11* and linked this to fluconazole resistance, pointing to the importance of aneuploidy to within-host evolution.

Beyond aneuploidy, a number of studies have identified clinical isolates capable of rapid adaption through hypermutation, largely linked to mutations in mismatch repair gene *MSH2* in *C. gattii* VGII (*C. deuterogattii*) (Billmyre et al. 2014, 2017) and *C. neoformans* var. *grubii* (Boyce et al. 2017, Rhodes et al. 2017a). Experimental measurements by Boyce *et al.* and Billmyre *et al.* (Billmyre et al. 2017, Boyce et al. 2017) demonstrated that deletion of *MSH2*, *MLH1*, and *PMS1* all produce hypermutator phenotypes, and deletion of *MSH2* specifically increases the rate of insertions and deletions in homopolymer tracts. This can increase the rate of drug resistance acquisition, particularly involving genes with long homopolymer tracts. Mutation of *PMS1* resulted in reduced virulence whereas mutation of *MLH1* and *MSH2* maintained fully virulent phenotypes (Boyce et al. 2017), supporting the hypothesis that hypermutator phenotypes can occur without a significant fitness tradeoff.

*Cryptococcus* microevolution has also been studied in the context of the laboratory strain *C. neoformans* var. *grubii* H99 (Janbon et al. 2014). This strain decreased in virulence after laboratory passaging and increased in virulence after passage in a rabbit model, comparing isolates from multiple laboratories (Janbon et al. 2014). Sequencing of three isolates exhibiting phenotypic differences revealed a frameshift in *LMP1* resulted in strain H99C being largely avirulent and infertile (Janbon et al. 2014). This illustrates the variation present in common laboratory isolates; understanding these differences and updating strain names to reflect this is critical for comparison of the genetic background of strains used in experiments across laboratories, as isolates may have diverged from wild type genotypes.

*Future directions leveraging technological advances* - As the above studies illustrate, genome sequencing of *Cryptococcus* is taking place at an increasing scale encompassing hundreds of isolates. As much of this data is being generated on a single platform, Illumina, combining and comparing data from different studies can be simplified. However, it is important to consider how differences in coverage level, library preparation chemistry, and sequencing bias could impact results from combined data sets (Rhodes et al. 2014); for example, sequencing bias could lead to under-representation of some regions of the genome, affecting analyses of copy number variation. Heterozygous isolates are also occasionally detected and require different protocols for variant identification and analysis (Rhodes et al. 2017b). In addition, such data re-use is potentiated by clearly documented metadata that typically includes the source of isolation and occasionally additional phenotypes measured experimentally.

These genomic studies rely on high quality reference assemblies and annotations. Current analysis of sequence data is based on comparisons to a small number of reference assemblies, which have benefitted from collaborative work to generate and curate their content. However, a single genome does not completely represent a species; gene gain and loss has been observed between isolates of *C. neoformans* var. *grubii* (Day et al. 2017, Rhodes et al. 2017b). In addition, most assemblies are not yet complete telomere to telomere representations of the genome, even for these reference genomes. Illumina data has been utilized to generate highly complete assemblies for additional isolates of *Cryptococcus*, with highest continuity where paired-end data from larger insert libraries is included; yet these assemblies do not represent complete genomes as they have many more scaffolds and contigs than chromosomes (Day et al. 2017, Rhodes et al. 2017b). Assembly of long reads generated by Pacific Biosciences or Oxford Nanopore Technologies may provide more contiguous assemblies, as they should help resolve repetitive regions including the centromeres and subtelomeres. However, targeted finishing effort may also be required to resolve some regions and achieve an end to end representation of these genomes.

Incorporation of additional genome-wide data sets have also provided another layer of annotation to reference genomes. The gene sets of the *C. neoformans* var. *grubii* (H99) and *C. neoformans* var. *neoformans (*JEC21) reference genomes have benefitted from RNA-Seq guided gene structure prediction (Janbon et al. 2014, González-Hilarion et al. 2016); this has improved the accuracy of splice site annotation in gene models and has also suggested the presence of large numbers of non-coding transcripts. RNA-Seq has also been used to update gene sets for each of the three main lineages of *C. neoformans* var. *grubii* and supported the prediction of lineage-specific genes (Rhodes et al. 2017a). Recent studies have begun to map modified bases across the *Cryptococcus* genome (Catania et al. 2017), and three dimensional genome mapping techniques utilizing sequencing to map higher order adjacency of genomic regions may also be applied in the near future. The integration of such diverse data sets into a more comprehensive model of the genome, and how it changes across time and varies within the population, will provide a more complete framework to study the evolution of these pathogens.

Increased population-level sequencing will also be required to further identify genetic mechanisms of virulence, drug resistance, and within-host adaptation. Genome-wide association studies require extremely large sample sizes to link variants to complex phenotypes, such as infectivity or virulence; while studies have begun to build this dataset (see Desjardins et al. 2017), further increases in sample size will enable further genetic insight. However small sets of isolates have the potential to link genotype to phenotype; for example, a comparing genomes for closely related isolates differing in the production of Titan cells identified stop codon mutations in the protein kinase A regulatory gene *PKR1*, for which prior work had demonstrated a role in Titan cell production (Choi et al. 2012, Hommel et al. 2017). Furthermore, while these genomic studies have examined individual isolates that are colony purified from clinical or environmental samples, metagenomic approaches could assist uncovering diversity within an individual host or site. For example, metagenomic sequencing of the population from serial isolates within a single patient over time would enable analysis of how mutations occur and become fixed. In sum, large-scale sequencing of both global populations and within single patients will continue to play critical roles in understanding the genetic basic of human virulence in *Cryptococcus*.
